# Structural evolution of the whole mitochondrial genome and phylogenetic inference in snakes (Squamata: Serpentes), including the undescribed mitogenome of the Brazilian endemic and critically endangered pitviper *Bothrops insularis*

**DOI:** 10.1590/1678-4685-GMB-2024-0196

**Published:** 2026-01-30

**Authors:** Igor Salles-Oliveira, José S.L. Patané, Milton Y. Nishiyama-, Maria José J. Silva

**Affiliations:** 1 Instituto Butantan (IB), Laboratório de Ecologia e Evolução (LEEV), São Paulo, SP, Brazil.; 2 Universidade de São Paulo (USP), Instituto de Biociências (IB), São Paulo, SP, Brazil.; 3 Hospital das Clínicas da Faculdade de Medicina da Universidade de São Paulo (HCFMUSP), Instituto do Coração (InCor), Laboratório de Genética e Cardiologia Molecular (LGCM), São Paulo, SP, Brazil.; 4 Instituto Butantan (IB), Laboratório de Toxinologia Aplicada (LETA), São Paulo, SP, Brazil.; 5 Instituto Butantan (IB), Núcleo de Bioinformática e Biologia Computacional (NBBC), São Paulo, SP, Brazil.

**Keywords:** Conservation, comparative genomics, ESU, golden-lancehead, Serpentes

## Abstract

Evolutionary analyses of mitogenomes have provided insights into species evolution and conservation. Studies on snakes revealed a higher diversity especially involving variation in tRNA clusters. However, despite the increase in assemblies in databases, the available information for Brazilian species remains scarce in mitogenomic surveys. Based on this, we sequenced and provided the first description of the mitogenome of *Bothrops insularis,* a critically endangered taxon. We also used the *B. insularis* assembly and 128 molecules available in databases up to March 2022 to explore rearrangements and evolution of the mtDNA under a phylogenomic perspective. Comparative analyses revealed 24 mitotypes due to rearrangements within four tRNA clusters and within control regions. Mitotype 1 (M1) and Mitotype 2 (M2) are restricted to Scolecophidia and M3 is highly distributed within Alethinophidia. In addition, the M3 is suggested as the most likely ancestral mitotype during the mitogenome evolution. Regarding *Bothrops*, we recovered the same mitotype for *B. jararaca* and *B. insularis*, which structure differs from other *Bothrops* species. Nucleotide variation suggests 1.5% divergence between *B. jararaca* and *B. insularis*. Therefore, we suggest that *B. insularis* can be considered an evolutionary significant unit, and the data generated herein can be valuable for insights into genome evolution and conservation.

## Introduction

The mitochondrial DNA composition and organization in metazoans have generally been considered as conserved, however advancements in sequencing technologies have improved the possibility of having more accurate studies on the structure of the molecule. Recent studies revealed that these variations could involve differences in gene order, composition, and length within and among genera, reflecting the intrinsic evolutionary process of each organism and offering valuable insights for phylogenomic, evolutionary and conservation studies (Macey et al., 1997; Gray, 2012; Bernt et al., 2013 a , b).

Snake mitogenome studies have revealed evolutionary changes involving gene order, nucleotide composition and different evolutionary rates. Early research described the origin of the light strand replication (OL) and the existence of variations in tRNA clusters, particularly in WAN-OL-CY and IQM regions (Seutin et al., 1994; Kumazawa and Nishida, 1995). Subsequently, Kumazawa *et al.* (1996) reported the existence of a duplicate control region encompassing conserved blocks (C-rich; Hairpins I, II and III; CoreTA; Repetitive Region I and II; CSB-I; and CSB-III) in Alethinophidian and changes involving the Proline-tRNA (P) within Viperidae. Later, studies clarified the structure of the complete mtDNA molecule as a typical circular genome, with 16 kbp encompassing 13 protein-coding genes (PCG), two ribosomal RNA (rRNA), 22 tRNA, one OL, and two control regions (CRI and CRII) (Kumazawa *et al.*, 1998; Dong and Kumazawa, 2005).

Further studies evinced the existence of 11 general structures (mitotypes) in snakes, differing mainly by tRNA rearrangements and presence/absence of non-coding regions. These findings suggested a higher diversity than previously proposed (Douglas et al., 2006; Jiang et al., 2007; Yan et al., 2008; Chen and Zhao, 2009; Oguiura et al., 2009; Qian et al., 2018). More recently, studies have discussed the evolution of associated control regions reinforcing the existence of conserved blocks and the repetitive nature of these regions, which highlights the importance of properly annotating these regions in comparative analyses (Xiaokaiti et al., 2022).

Despite these advances, Brazilian species remain underrepresented in mitogenomic surveys due to a lack of complete mtDNA genomes. Recent sequencing efforts have begun to address this gap, particularly for the genus *Bothrops* (Almeida et al., 2016), which is the genus that causes the most snakebites in the country (Silva et al., 2023). The first complete mitogenome of a Brazilian species, *Bothrops jararaca*, revealed a novel rearrangement, suggesting that Brazilian snakes could harbor a hidden mitogenomic diversity (Almeida *et al.*, 2016).

*Bothrops* is a genus widely distributed throughout Latin America, currently including 48 species, according to The Reptile Database (2024), known for medical and ecological relevance (Campbell and Lamar, 2004). This genus can be subdivided into seven major groups based on morphological and genetic characters (Carrasco et al., 2023). Among these groups, the *Bothrops jararaca* group stands out from a conservation perspective, due to the presence of five endemic island species of the Brazilian coast, three of which are classified at some level of extinction risk according to the International Union for Conservation of Nature - IUCN (Barbo et al., 2022). 

Phylogeographic studies have proposed that their isolation process occurred 2.37 million years ago, probably driven by sea-level fluctuation and cycles of geographic isolation (Barbo et al., 2022). This, together with other genetic processes (such as genetic drift) might lead to unique mitogenome structures. 

In this study, we focus on *Bothrops insularis*, an endemic critically endangered species from the Ilha da Queimada Grande (Amaral, 1921; Brasil, 2022), which represents a flagship for conservation (Salles-Oliveira et al., 2020) with the goal of sequencing the total mitogenome, comparing its structure and evolution with other *Bothrops* species and other snake representatives. 

## Material and Methods

### *Bothrops insularis* mitogenome sequencing

Ventral scales were obtained from three representatives of *B. insularis* (two females and one male) and the total genomic DNA was extracted using the DNeasy Blood & Tissue Kit (Qiagen), improving the standard protocol by adding 20 µL of 20% Sodium dodecyl sulfate (SDS) during the tissue lysis phase. Then, samples were diluted to a volume of 50 µL with 30 to 50 ng/µL.

Diluted solutions were sent to the Centro de Genômica Funcional da Escola Superior de Agricultura “Luiz de Queiroz” - ESALQ/USP - for the construction of an enriched DNA library for each sample, and posterior sequencing. Libraries were constructed using the Nextera DNA Flex Library Prep Kit following the manufacturer’s instructions. High-throughput paired-end sequencing was performed in one lane in the Illumina Hiseq 2500 sequencer set for 2 read x 150 bp reads, using the HiSeq SBS v4 Kit.

Library samples were demultiplexed and the raw fastq paired-end read files obtained from the sequencer with Quality-based (Q) > 30, processed by the CASAVA library v. 1.8.2. Raw reads were trimmed and filtered for contaminants (PhiX, *Pichia pastoris* and *Escherichia coli*) using the software Bowtie 2 v.2.2.3 (Langmead and Salzerberg, 2012). Further, quality filtering was performed using the Trimmomatic software v.0.36 (Bolger et al., 2014) to remove adapters, to trim the 5′ and 3′ ends with mean quality score below 25 (Phred+33), and to discard reads shorter than 30 bp after trimming.

*B. insularis* mtDNA size was estimated by *K-*mer analyses of the 150bp Illumina paired-end reads, using the program GenomeScope which applies mixed negative binomial model to grant greater flexibility in genome size estimation (Vurture et al., 2017). *B. insularis* mitogenome was assembled using two methodologies: (i) *de novo* assembly with ABYSS (k-mer = 75), and (ii) using a reference guided assembly with CLC Genomics Workbench software v.11.0.1 - Qiagen -, aligning the *B. insularis* reads against the *B. jararaca* mitogenome and extracting the resulting consensus sequence.

### mtDNA from databases

Snake mtDNAs were obtained from the National Center for Biotechnology Information (NCBI) database during the period of 01-07-2020 to 01-03-2022. The selected sequences were RefSeq preferably, except in those cases where the sequence was unique to the species. A total of 128 sequences were obtained from these databases and used in this study (Table S1).

### mtDNA (re)annotation and structural comparisons

The 128 mtDNA sequences accessed from GenBank plus the *B. insularis* mtDNA sequenced in this study were reannotated to predict tRNA, rRNA and protein-coding genes using a multiple approaches: (i) an initial prediction using three software tools - MITOS 2 (Donath et al., 2019), tRNAScan-SE v.2.0 (Chan et al., 2021) and MitoFinder (Allio et al., 2020) - which diverge on algorithm sensitivity and accuracy (Jühling et al., 2012); (ii) alignment by BLAST of each region against the NCBIdatabases (NT/NR; TSA; RefSeq RNA; and HTGS) and the Universal Resource Proteins - UNIPROT; (iii) comparing each non-annotated region against each tRNA of the species by using the function map to reference implemented in the program Geneiousv.7.1.7 (Kaerse et al., 2012) with default parameters; and (iv) a comparison with corresponding gene sequences from other snake mitogenomes and manual inspection of their nucleotide composition. Then, for each species the annotations were compared and compiled in a single dataset, which was used for further analyses.

Control regions were annotated by mapping the conserved blocks described by Kumazawa et al. (1996), using the “map to reference” function implemented in Geneious v.7.1.7 with default parameters. Subsequently, sequences were aligned within genera, then family and superfamily with the align algorithm ClustalW (Thompson et al., 1994) and manual curation was performed to confirm the conserved block existence, as done by Xiaokaiti et al. (2022).

After all, mtDNA data were manually curated in Geneious v.7.1.7 comparing each sequence regarding the structure and gene order taking into account the (re)annotation results obtained from the previous steps. Readers interested in obtaining the alignments can contact the corresponding author. 

### mtDNA analyses within the genus *Bothrops*

*B. insularis* mitogenome was compared against available mitogenomes from three representatives of the genus *Bothrops*: *B. jararaca* (Almeida et al., 2016), *B. diporus* (GenBank accession no. NC_039649.1) and *B. pubescens* (GenBank accession no. NC_039648.1). *Bothrops* representative mitogenomes were compared using Geneious v.7.1.7 based on the structure and gene order. Nucleotide composition of each mitogenome was inferred by MEGA X (Kumar et al., 2018). CG and AT skew were calculated as described by Perna and Kocher (1995). Mitogenomes schemes and CG skew were drawn using the web server CGView (Stothard and Wishart, 2005). 

### Phylogenetic analyses

Phylogenetic analyses were performed using a matrix composed of the 13 protein-coding genes and the two rRNA subunits of the 129 snake mitogenome sequences reannotated. *Chelodina oblonga* (GenBank accession no. NC_037387), *Crocodylus acutus* (GenBank accession no. NC_015647), *Plestiodon egregious* (GenBank accession no. NC_000888), *Varanus salvator* (GenBank accession no. NC_010974), *Chamaeleo chamaeleon* (GenBank accession no. NC_012427) and *Iguana iguana* (GenBank accession no. NC_002793) were selected as representatives of Squamata clades and used as the outgroup. 

Genes and rRNA sequences were subdivided using Geneious, and aligned individually in AliView (Larsson, 2014) by using the Muscle (Edgar, 2004) algorithm. All alignments were manually checked to minimize alignment errors. Each alignment was divided into a unique matrix based on the codon position using FasParser v. 2.10.0 (Yan-Bo, 2017). Then, these unique matrices were concatenated to create a super matrix, and each partition was annotated using FASCONCAT.PL (Kück and Meusemann, 2010). The best-fit evolutionary model for each partition was inferred by the MODELFINDER algorithm (Kalyannamoorthy et al., 2017) within IqTree v.1.6.12 (Nguyen et al., 2015).

Phylogenetic trees were constructed by Maximum Likelihood (ML) and Bayesian Inference (BI) methods. ML was implemented in IqTree v.1.6.12 employing the evolutionary models and partitions obtained by MODELFINDER, and 1,000 rapid bootstrap replicates were used to infer UFBoot2 (Hoang et al., 2018) support values and the best supported ML tree. BI method was implemented in MRBAYES v.3.2 (Ronquist et al., 2012) using the same models as in ML. Prior to analyses, Dirichlet prior parameter was calculated as described in the MRBAYES manual, by using the package Ape (Paradis and Schliep, 2019) implemented in Rto assess tree length, internal branch lengths, and external branch lengths. Subsequently, 20,000,000 generations were performed in two runs of four independent Markov Chain Monte Carlo (MCMC) chains, sampled every 1,000 generations, with burn-in of 30%. Bayesian convergence was investigated with the software TRACER v.1.7.1. ML and BI trees were visualized in FigTree v.1.4.3.

### Ancestral character state reconstruction

To address the evolutionary pathway of the mitogenomes we conducted an ancestral character state reconstruction using the R packages Ape (Paradis et al., 2004) and phytools (Revell, 2024). Briefly, each mitotype was assigned as a discrete character and compiled in a matrix. This matrix and the ML tree retrieved from the previous analyses were used as input. The estimate of each ancestral character was recovered applying the ML method and the model ER (default parameters).

### Ethics Approval

Sample collection was performed under license and authorization of the Ethics Committee on the Use of Animals from Instituto Butantan (CEUAIB - protocol number: 15131705/18) and under the license of the Sistema de Autorização da Informação da Biodiversidade (SISBIO - protocol number: 63295/12). The completion of this project and its methods were performed under license and authorization of the Ethics Committee on the Use of Animals from Instituto Butantan (CEUAIB - protocol number: 3124270671/17). Also, the authors declare that all experiments and methods were performed in accordance with relevant guidelines and regulations and address the ARRIVE guidelines for the reporting of animal experiments.

## Results

### *B. insularis* mitogenome

A total of 570,582 Illumina paired-end reads (2 x 101 bp), amounting to 55,249,782 bp effectively used in the mitochondrial assembly, resulting in an average coverage depth of approximately 3,188x. The *de novo* assembly recovered a circular molecule of 16,606 bp (not shown), while the reference-guided assembly recovered a molecule of 17,523 bp. Both assemblies recovered the same topology and gene order. However, the *de novo* assembly did not recover the Control Region I (CRI) segment, probably due to the limitation of the short reads sequencing in assigning repetitive regions, as we will discuss below. Given this suggested potential sequencing artifact, we will describe our results based on the reference-guided assembly.

**Figure 1 -:**
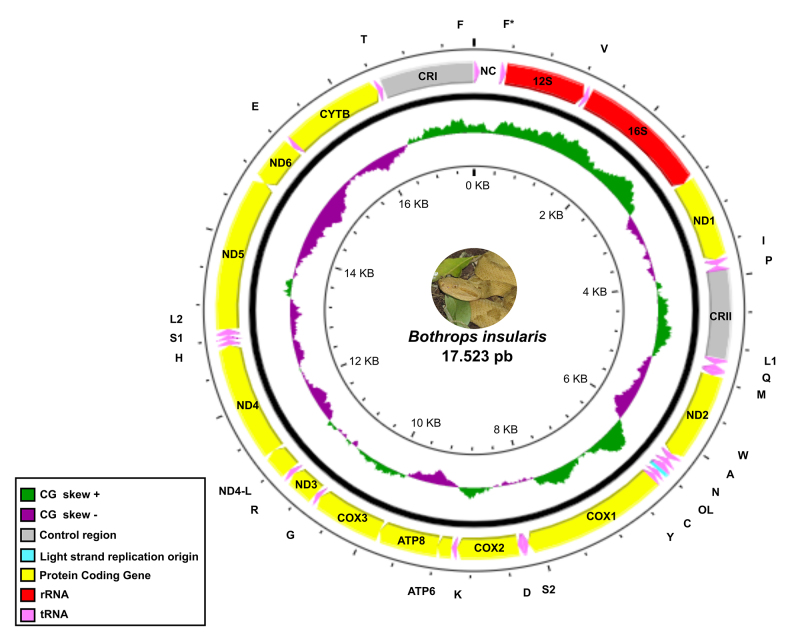
*Bothrops insularis* mitogenome scheme. + strand and - strand are indicated by arrows’ directions. Mitogenome components were highlighted by colors: Dark green = CG Skew +; Gray = Control Regions; Light Blue = Light Strand Replication Origin; Pink = tRNA sequences; Purple = CG Skew -; Red = rRNA subunits (12S and 16S) sequences; Yellow = Protein-Coding Genes.

The *Bothrops insularis* mitogenome was identified as a circular molecule with a length of 17,523 bp (Figure 1), encompassing 13 PCG, 23 tRNA, two control regions (CRI and CRII), two rRNA, one light strand replication origin, and one non-coding region. Twenty-nine components are encoded on the forward strand and nine on the reverse one (Figure 1). Nucleotide composition was revised for each strand to correctly assign the heavy (H) and the light (L) strand. The forward strand is composed of 31.9% adenine, 24.4% thymine, 30.3% cytosine and 13.3% of guanine; the reverse strand is composed of 24.4% adenine, 31.9% thymine, 30.3% guanine and 13.3% cytosine. Based on the higher guanine content, and following the buoyant density, we designed the reverse strand as the heavy strand, and the forward strand as the light strand. Accordingly, the heavy (H, +) and the light strand (L; -) comprises nine and 28 components, respectively (Table S2). Among the 23 tRNA sequences recovered (Figure S1), 21 were unique and two were associated to the Phenylalanine-tRNA (F). Secondary structures of both tRNA-F share 83.1% of similarity, with the second copy - tRNA-F (F*) - differing by the absence of the arm of TΨC stem-loop.

### Comparative analyses of the mitogenomes of the genus *Bothrops*

**Figure 2 -:**
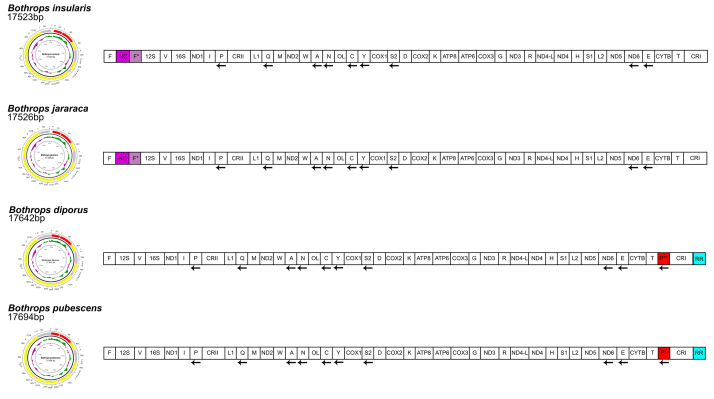
Mitogenomes of representatives of the genus *Bothrops*: *B. insularis*, *B. jararaca*, *B. pubescens*, and *B. diporus*. Additionally, to the circular molecule, a linear diagram is shown to improve the components representation. Mitogenomic components that diverge among the species are highlighted by colors. Genes, tRNAs and rRNAs names are abbreviated. **Genes:** ND1 = NADH dehydrogenase subunit 1, ND2 = NADH dehydrogenase subunit 2, ND3 = NADH dehydrogenase subunit 3, ND4 = NADH dehydrogenase subunit 4, ND4-L = NADH dehydrogenase subunit 4L, ND5 = NADH dehydrogenase subunit 5, ND6 = NADH dehydrogenase subunit 6, COX1 = Cytochrome C oxidase subunit 1, COX2 = Cytochrome C oxidase subunit 2, COX3 = Cytochrome C oxidase subunit 3, ATP8 = ATP synthase subunit 8, ATP6 = ATP synthase subunit 6, CYTB = Cytochrome B; **tRNAs:** F = phenylalanine, F* = Duplicate F, V = Valine, I = Isoleucine, I* = Duplicate I, P = Proline, P* = Duplicate P (partial sequence), P** = Duplicate P (complete sequence), L1 = Leucine1, Q = glutamine, M = Methionine, W = Tryptophan, A = Alanine, N = Asparagine, N*= Duplicate N, C = Cysteine, Y = Tyrosine, S2 = Serine 2, S2* = Duplicate S2, D = Aspartic Acid, D* = Duplicate D, K = Lysine, G = Glycine, R = Arginine, R = Histidine, S1 = Serine 1, L2 = Leucine 2, E = Glutamic Acid, T = Threonine , T* = Duplicate T. **rRNAs:** 12S = Subunit 12S, 16S = Subunit 16S; **Other features:** NC = Non-coding region, RR = Repetitive region, CRI = Control region I, CRII = Control region II.

Structure and comparative analyses of the mitogenomes of *B. insularis*, *B. jararaca*, *B. diporus,* and *B. pubescens* suggest a highly conserved distribution of these molecular components (Figure 2). Nucleotide composition was conserved among the analyzed *Bothrops* species, with small divergences (around 0.1 - 0.5%), likely due to their close evolutionary relatedness.

Despite this overall similarity, some divergences were observed: (i) the duplicated tRNA-F* and NC region prior the rRNA subunit 12S in *B. insularis and B. jararaca*, with NC segments alignment revealing similarities (46 to 48%) with the end of the CRI; and (ii) the duplicated Proline-tRNA (P**) prior the CRI in *B. diporus* and *B. pubescens*, which nucleotide sequence were highly maintained and complete when compared to other Viperidae species. 

### Phylogenomic inference based on mtDNA

**Figure 3 -:**
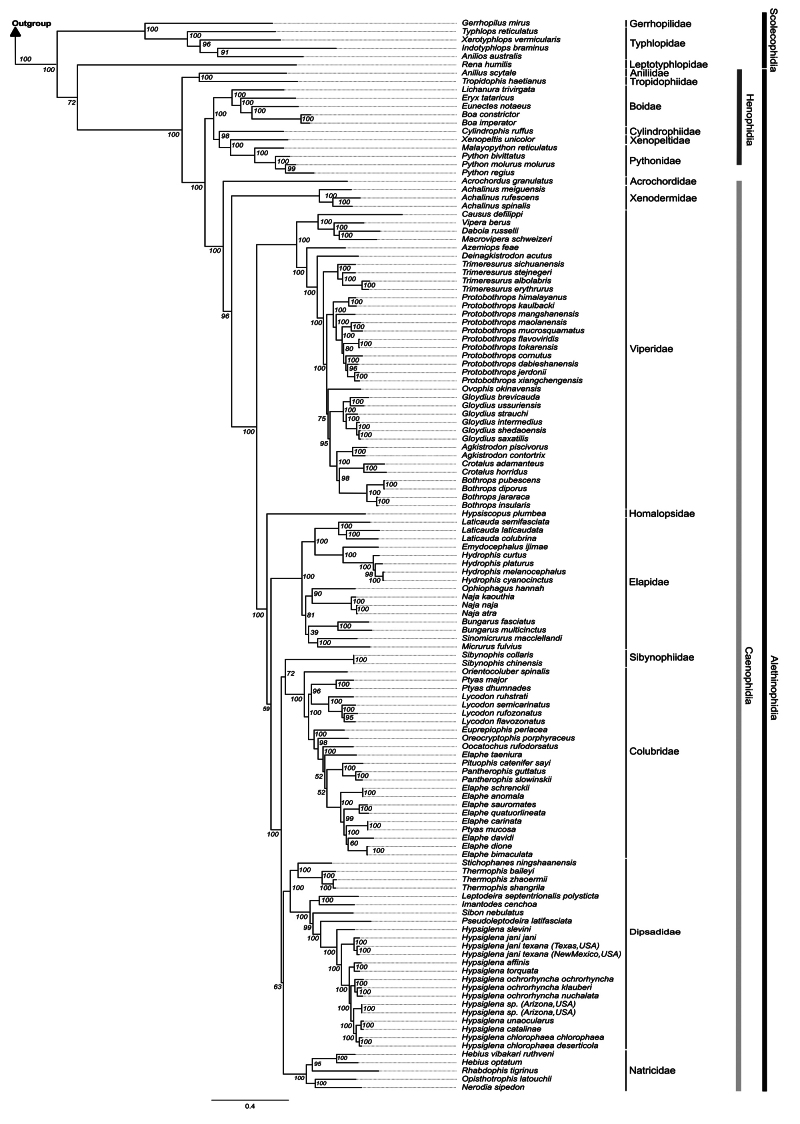
Maximum Likelihood phylogenetic tree based on 13 Protein-coding genes and the two mitochondrial rRNA subunits (12S and 16S). Numbers near nodes are bootstrap values.

Phylogenomic analyses based on BI and ML recovered very similar topologies, except for divergences in low supported clades (Elapidae, Sibynophiidae, Colubridae, Dipsadidae, and Natricidae) which were recovered as a polytomy in BI (not shown). Due to the overall agreement in both phylogenies, phylogenetic discussion will be based on the ML topology (Figure 3).

Scolecophidia was recovered as a paraphyletic group (ML: 100%), whereas Alethinophidia was monophyletic (ML: 100%). The clade composed of Gerrhopilidae and Typhlopidae (ML: 100%) was recovered as the sister group to the other taxa (ML: 100%). Leptotyphlopidae were recovered as the sister group to Alethinophidia (ML: 72%).

Within Alethinophidia two major clades were recovered: Henophidia (as a paraphyletic group) and Caenophidia (as a monophyletic group). The clade composed of Aniliidae and Trophydophiidae (ML: 100%) were recovered as the sister group to a clade composed of the other henophidians (ML: 100%) and a clade composed of caenophidians (ML: 100%).

Within Caenophidia, the branch composed of Acrochordidae diverges firstly (ML: 100%), followed by Xenodermidae (ML: 96%) which is the sister to Viperidae which is the sister to the other snakes (ML: 100%). Homalopsidae is the sister group to the other snakes (ML > 96%), although Elapidae was recovered as the sister group to the remaining families with ML: 59%. The remaining families were recovered in two distinct clades (ML: 100%): the first, composed of Sibynophiidae and Colubridae (ML: 72%), and the second one, composed of Dipsadidae and Natricidae (ML: 63%). Although these two clades showed low support values, the values recovered for each family within these clades were high (ML: 100%). 

### Snake mitogenome characterization and evolution

**Figure 4 -:**
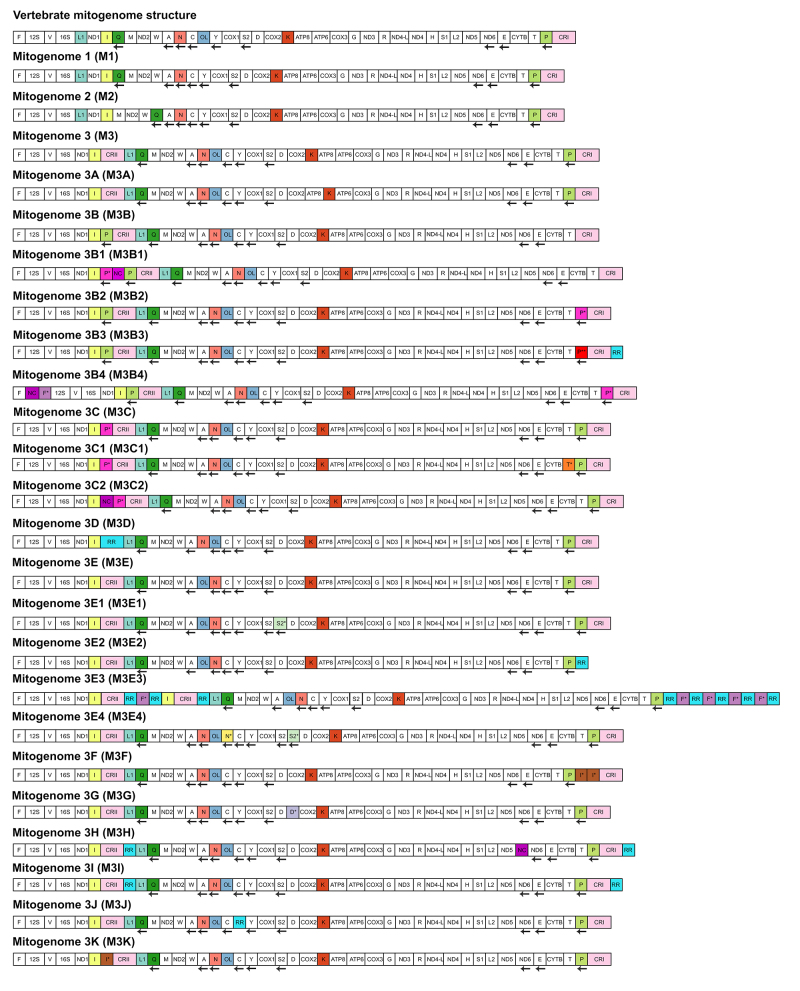
Components arrangement of the mitogenomes of snakes. Mitotypes are named as M1, M2, M3, and M3A to M3K. H-strand and L-strand are indicated by the absence or presence of an arrow, respectively. Mitogenomic components involved in rearrangements are highlighted with different colors. **Genes:** ND1 = NADH dehydrogenase subunit 1, ND2 = NADH dehydrogenase subunit 2, ND3 = NADH dehydrogenase subunit 3, ND4 = NADH dehydrogenase subunit 4, ND4-L = NADH dehydrogenase subunit 4L, ND5 = NADH dehydrogenase subunit 5, ND6 = NADH dehydrogenase subunit 6, COX1 = Cytochrome C oxidase subunit 1, COX2 = Cytochrome C oxidase subunit 2, COX3 = Cytochrome C oxidase subunit 3, ATP8 = ATP synthase subunit 8, ATP6 = ATP synthase subunit 6, CYTB = Cytochrome B; **tRNAs:** F = phenylalanine, F* = Duplicate F, V = Valine, I = Isoleucine, I* = Duplicate I, P = Proline, P* = Duplicate P (partial sequence), P** = Duplicate P (complete sequence), L1 = Leucine1, Q = glutamine, M = Methionine, W = Tryptophan, A = Alanine, N = Asparagine, N*= Duplicate N, C = Cysteine, Y = Tyrosine, S2 = Serine 2, S2* = Duplicate S2, D = Aspartic Acid, D* = Duplicate D, K = Lysine, G = Glycine, R = Arginine, R = Histidine, S1 = Serine 1, L2 = Leucine 2, E = Glutamic Acid ,T = Threonine , T* = Duplicate T. **rRNAs:** 12S = Subunit 12S, 16S = Subunit 16S; **Other features:** NC = Non-coding region, RR = Repetitive region, CRI = Control region I, CRII = Control region II.

**Figure 5 -:**
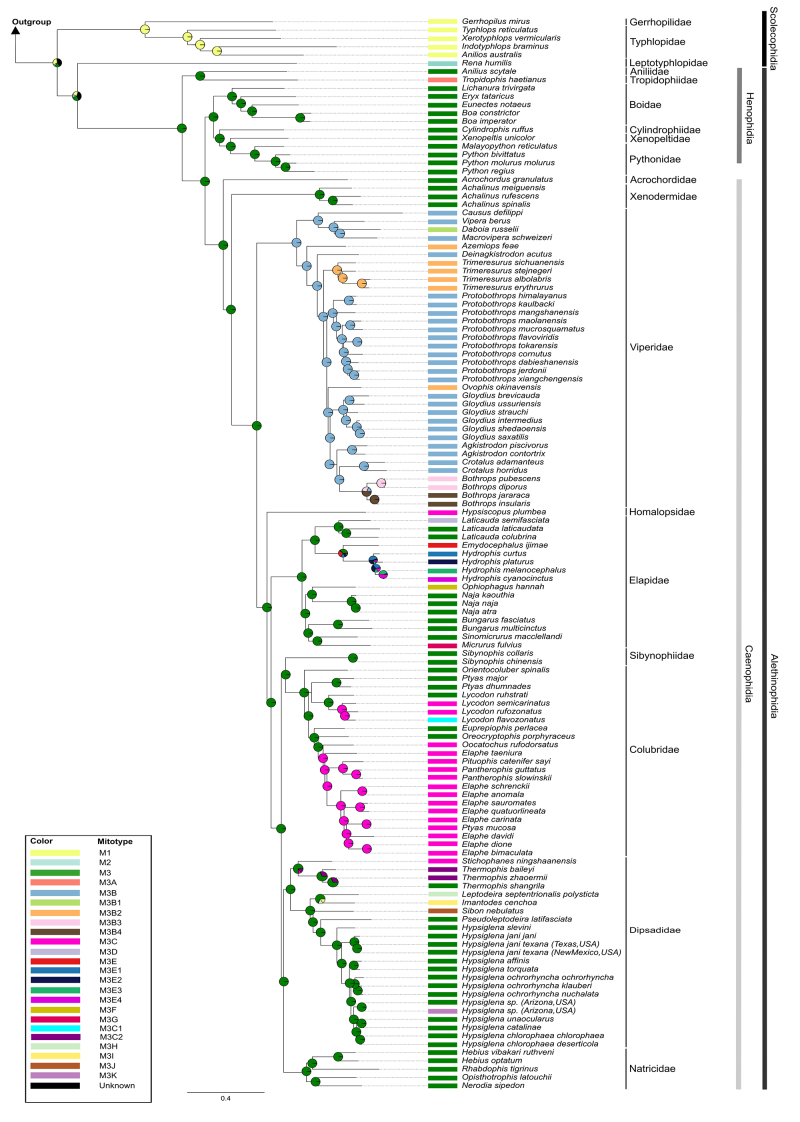
Snake mitogenome evolution under a phylogenomic perspective. Mitotypes M1 to M3K represent the same types as those shown in Figure 4. Pie chart at each node represents the most probable mitotype of each ancestral node.

Regarding the structural diversity and evolution, 24 mitotypes were recovered (Figure 4), for which the phylogenetic pattern was also reconstructed (Figure 5): (i) mitotypes 1 (M1) and 2 (M2) were found in Scolecophidia albeit M2 is restricted to Leptotyphlopidae, both mitotypes were diagnosed by rearrangements involving the OL region and/or the Q-tRNA; (ii) M3 is highly distributed in Henophidia and Caenophidia, and it was diagnosed by rearrangements involving the CRII and the L1-tRNA; (iii) divergent structures from M3 were related to Caenophidian specific families (M3A - Aniliidae: rearrangements involving the K-tRNA; M3B and its variations - Viperidae: rearrangements involving the P- and F-tRNAs adjacent to control regions; subfamilies (M3E and its variations - Hydrophiinae: rearrangements involving the N- and S-tRNAs, presence or absence of control region, and presence of repeat motif at 3’ end of the control region); or species (M3F - *Ophiophagus Hannah*) // M3K - *Hypsiglena jani texana* (New Mexico, USA): rearrangements involving the I-tRNA; M3G - *Micrurus fulvius*: rearrangements involving the D-tRNA; M3C1 - *Lycodon flavozonatus*: rearrangements involving the T-tRNA; M3H - *Leptodeira septentrionalis polysticta //* M3I - *Imantodes cenchoa* // M3J - *Sibon nebulatus*: rearrangements involving NC and repetitive regions at 3’ end of the control region, between the C-tRNA and Y-tRNA or between the genes ND5 and ND6; except for the mitotype M3C which was recovered in different families (Colubridae, Dipsadidae, and Homalopsidae): rearrangements involving the P-tRNA. 

Ancestral character state reconstruction analyses (Figure 5) recovered the M3 as the most likely ancestral mitotype within Alethinophidia. The ancestral state between the Scolecophidia and Alethinophidia were not obtained due to the small probability of each mitotype, reinforcing the possibility of independent origins of the mitotype 1, 2 and 3. Additionally, other variants within Alethinophidia were restricted to clade-specific (for example *Hydrophiinae*) or to species-specific (for example *Trimeresurus* ssp*.*). 

**Figure 6 -:**
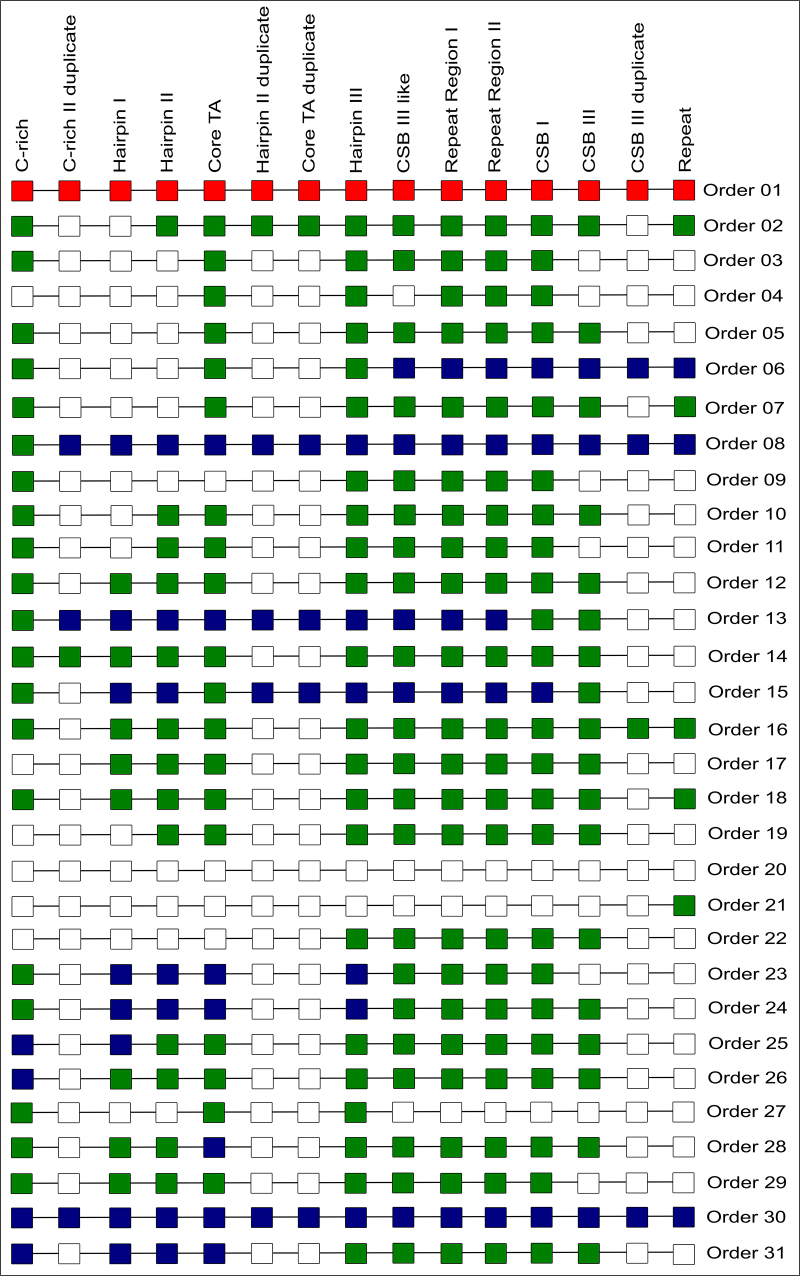
Control regions structures found in this study. White square = absence of the conserved region on the available sequence; Blue square = incomplete sequence with N or Gaps which the presence or absence of the conserved region could not be checked; Green square = presence of the conserved region on the available sequence. Red square = incomplete sequence due to the lack of the sequencing of the control region.

**Figure 7 -:**
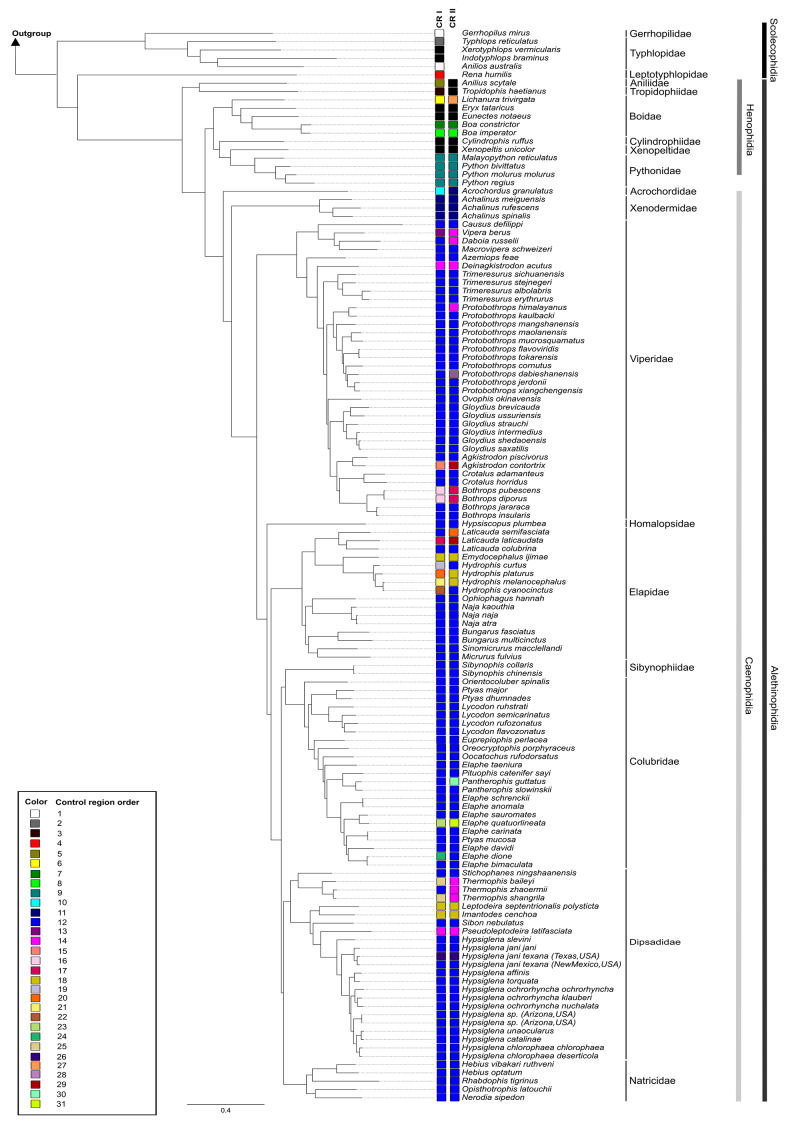
Snake control region evolution under a phylogenetic perspective. Control regions order 1 to 31 represent the same types as those shown in Figure 6. **CR1** = Control region 1. **CR2** = Control region 2.

Beyond the gene order and the variation presented above, conserved blocks within the control region segments were identified (Figure 6). We found that C-rich, coreTA, Hairpin III, Repeat Region I, Repeat Region II and CSB-I regions are highly conserved among control regions. However, Hairpin I, Hairpin II and CSB-III region were highly variable, leading us to refrain from characterizing these regions on Henophidia specimens, after the alignment and manual curation due to possibility of erroneous identification. Based on these findings, two major patterns of control region structure were identified (Figure 7): (i) a structure lacking hairpins I and II, and CSB-III for Scolecophidia and Henophidia; and (ii) a structure containing hairpin I and II, and CSB-III for Alethinophidia is the prevalent structure among the mitogenomes analysed.

Duplication of some conserved blocks and repetitive regions were observed in few species (Figure 7). In Scolecophidia: *Typhlops reticulatus* has (i) a repeat with 152 bp (motif of 76 bp repeated two times *in tandem*) at the initial part of its control region that encompasses the hairpin II (39 bp) and core TA (16 bp) sequences, and (ii) a repeat with 255 bp at the final part of its control region (motifs of 106 bp repeated two times *in tandem* followed by an incomplete motif of 41 bp repeated two times *in tandem*, and an incomplete motif of 67 bp). In Henophidia: *Boa constrictor* has a repeat with 882 bp (motifs of 98 bp repeated nine times *in tandem* followed by an incomplete motif of 19 bp) at the final part of its control region I and II. It’s worth mentioning that *B. imperator* has a similar pattern, but we refrain to characterize it due to the low quality and the existence of gaps on the control region segments of its mitogenome available. In Viperidae: (i) *Bothrops diporus* and *Bothrops pubescens* have a repeat with 538 bp and 622 bp, respectively (motifs of 269 bp repeated two times *in tandem* and 311 bp repeated two times *in tandem* in *B. diporus* and *B. pubescens*, respectively) at the final part of their control region I that encompasses the CSBIII segment, (ii) *Daboia ruselli* has a repeat with 82 bp (motif of 41 bp repeated two times but interspaced) at the initial part of the control region II; (iii) *Deinakgistrodon* acutus has a duplication of the C-rich segment (26 bp) at the initial part of the control region II; (iv) *Protobothrops himalayanus* has a repeat with 168 bp (motifs of 84 bp repeated two times *in tandem*) at the initial part of the control region II that encompasses the C-rich segment (23 bp); (v) *Vipera berus* has a repeat with 120 bp (motif of 60 bp repeated two times *in tandem*) at the initial part of the control region II that encompasses the C-rich (24 bp) and the initial segment of Hairpin I (9 bp). In Dipsadidae: (i) *Imantodes cenchoa* has a repeat of 1,432 bp (expanded motif of 186bp, followed by an incomplete motif of 177 bp, and the complete motif of 179 bp repeated eight times *in tandem*) and of 2,966 bp (incomplete motif of 175 bp, followed by a complete motif of 179 bp repeated 15 times *in tandem*, followed by an incomplete motif of 106 bp) at the final part of the control region I and II, respectively; (ii) *Leptodeira septentrionalis polysticta* has a repeat of 3,852 bp (motif of 115 bp repeated 33 times in tandem, followed by an incomplete motif of 57 bp) and of 1,896 (motif of 115 bp repeated 16 times *in tandem*, followed by an incomplete motif of 56 bp); (iii) *Pseudoleptodeira latifasciata* has a repeat of 82 bp (motif of 41 bp repeated two times interspaced) and of 132 bp (motif of 66 bp repeated two times *in tandem*) at the initial part of the control region I and II, respectively, that encompasses the C-rich segment (21 bp); and (iv) *Sibon nebulatus* has a repeat of bp (motif of 214 bp repeated 24 times *in tandem*, followed by an incomplete motif of 112 bp) *Thermophis baileyi* and *T. zhaoermii* have a repeat of 40 bp repeated two times interspaced) at the initial part of the control region II that encompasses the C-rich segment (21 bp). 

Repeats in *Emydocephalus ijimae, Hydrophis melanocephalus* and *H. platurus* were previously described by Xiaokaiti et al. (2022) and, therefore, were not explored or discussed herein.

## Discussion

### *Bothrops* mitogenomes characterization and evolution

We present the first complete and structurally annotated mitochondrial genome of the critically endangered snake, *B. insularis*, which was reconstructed and described herein based on reference-guided assembly. Although the two *de novo* and reference-guided assemblies recovered the same topology and overall gene order, the *de novo* approach failed to recover the CR1. This absence could be associated with the limitation of short-read sequencing (as performed herein) to solve repetitive regions delimitation, as previously discussed by Formenti et al. (2021), who found the absence of complete or partial segments of control regions in *de novo* assemblies based on short-reads of various taxa. 

In our *de novo* assembly, we recovered a 94 bp region downstream the tRNA-T and a 47 bp segment upstream the first tRNA-F. Alignments of these two assemblies indicated that these regions correspond to the initial and terminal segments of CRI, respectively, on the reference-guided assembly. Considering this, and (i) the consistent presence of both CR regions on all the genome references for *Bothrops* and relatives’ species available, and (ii) the higher similarity (99.5%) among the control regions (I and II) recovered on assemblies, we could suggest that the lack of CRI on the *de novo* assembly would be more likely a sequencing limitation and artifact. Long-read sequencing approaches will be needed to confirm or refute the existence of this segment.

*B. insularis* mitochondrial DNA molecule is similar to the mtDNA of *B. jararaca* (Almeida et al., 2016) but differs in gene order and structure (presence or absence of components) when compared to *B. diporus* and *B. pubescens*. These divergences are associated to duplication of mitogenomic regions, such as: (i) a non-coding region (NC) followed by a duplicate tRNA-F* in *B. insularis* and *B. jararaca*; and (ii) a duplicate tRNA-P* and the final of the CR region in the CR I in *B. pubescens* and *B. diporus*.

Duplication of tRNA has been proposed for many taxonomic groups (see Jühling et al., 2012), which may vary in arrangements and size, involving overlapping tRNAs (Jühling *et al.*, 2012; Qian et al., 2018). The major hypothesis for the origin of duplicate regions in vertebrates is associated with tandem duplication and random loss (TDRL) process followed by the translocation of these duplicates or the original segment (Macey et al., 1997).

**Figure 8 -:**
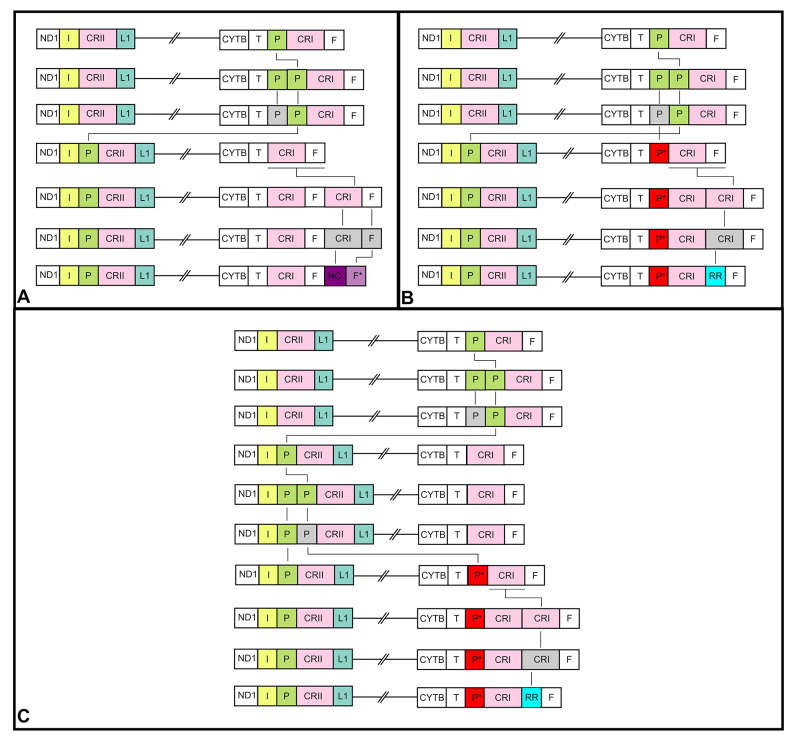
Mitogenome evolution pathway of *Bothrops* representatives. **A**: Evolutionary scenario of the MB34 mitotype. **B**: Evolutionary scenario of the MB3B mitotype with the relict tRNA-P from ancestral mitogenomes. **C**: Evolutionary scenario of the MB3B mitotype with two TDRL events. Mitogenomic components involved in rearrangements are highlighted with different colors. **Genes:** ND1 = NADH dehydrogenase subunit 1. CYTB = Cytochrome B; **tRNAs:** White F = phenylalanine. Gray F = modified or lost tRNA-F. I = Isoleucine. P = Proline. Gray P = modified or lost tRNA-P. P** = Duplicate P (complete sequence with anticodon recognition), L1 = Leucine (UAC). T = Threonine. **Other features:** NC = Non-coding region.

In *Bothrops* mitogenomes these might be observed by the similarity of the duplicated regions and tRNAs. In *B. insularis* and *B. jararaca* this process may involve the duplication of the final segment of the CRI and the tRNA-F in tandem after the original tRNA-F (Figure 8 A ). In *B. diporus* and *B. pubescens* this process might involve the duplication of the final segment of the CRI (bp) tandemly (Figures 8 B  and 8C), and the duplication of the tRNA-P which might be a relict segment of Viperidae ancestral mitogenome or a re-duplication process on these species (Figures 8 B  and 8C). Despite that, functional analyses of both duplicate tRNAs (F* and P*) could be tested to provide insights into the usage of these molecules and their importance in each mitotype.

It is worth mentioning that both mitotypes mentioned above are restricted to the genus *Bothrops*. Each mitotype might be exclusive for each *Bothrops* group (M3B3 - *B. neuwiedi* group; and M3B4 - *B. jararaca* group). However, further sequencing studies encompassing more sequences and other species belonging to the genus are necessary to test this hypothesis, enabling the correction of potential sequencing error and improving understanding of the mitogenomic evolutionary pathway on the genus. 

### Snake mitogenome evolution: Perspectives and insights

The comparative analyses using 129 mitogenomes of snakes, distributed in 18 families, allowed the inclusion of 64 species (including the *B. insularis* data) and four families in the snake mitogenome evolution scenario. The improvement in new families was due to: (i) use of the taxonomic proposition suggested in the phylogenomic revision of the Colubroidea superfamily which was divided in 18 families (Zaher et al., 2019); and (ii) the inclusion of a new specimen of the family Gerrophilidae when compared to the last revision performed by Qian et al. (2018).

The phylogenetic analyses partially recovered most evolutionary relationships found for Squamata in phylogenomic studies (for example, Zheng and Wiens, 2016 - used 52 genes and 4162 species; and Zaher et al., 2019 - 6 mitochondrial and 9 nuclear genes, and 1278 species), however some divergences should be highlighted. The first one is related to the Scolecophidia paraphyly that has already been recovered (Miralles et al., 2018; Qian et al., 2018), which disagrees with the monophyly found by Zheng and Wiens (2016). Once again it is worth mentioning that the paraphyletic clades recovered herein (Clade 1: Gerrophilidae and Typhlopidae; Clade 2: Leptotyphlopidae) differ from the ones recovered previously (Clade 1: Leptotyphlopidae, Gerrophilidae, Xenotyphlopidae and Typhlopidae; Clade 2: Anomalepididae; Miralles *et al.*, 2018). Moreover, our analysis recovered Natricidae as the sister clade to Dipsadidae, whereas Natricidae was recovered as the sister clade to (Sibynophiidae + Colubridae) by Zaher *et al.* (2019). Both disagreements might be attributed to the mtDNA genes representing a single non-recombining haplotypic molecule, thus not capturing the full set of independent gene topologies present in the phylogenomic studies that uses nuclear and mitochondrial genes. Another possibility is that these studies have different numbers of species compared due to the absence of the whole mtDNA. These differences could be resulting in different topologies of the trees, and therefore, this incongruence is still open to be investigated.

Aside from the phylogenetic analyses, comparative analyses focusing on gene order and structure allowed the recognition of 24 mitotypes within snakes. Although some of these had been previously described (Qian et al., 2018; Xiaokaiti et al., 2022), others were revised after our reannotation process (e.g. the lack of recognition of the tRNA-P* in the genus *Elaphe*, which had been suggested for *E. scherenckii* by Park et al. (2022) and herein after manual curation process it was confirmed in all *Elaphe* species).

Regarding mitogenome diversity and evolution, ancestral state reconstruction performed herein recovered M3 as the prevalence on the common ancestors in snakes’ evolution, with variants occurring in few clades (M1 and M3B) and with convergent episodes (M3C), with no clearly state being confidently assigned within Scolecophidia and Alethinophidia, which reinforces the hypothesis proposed by Qian et al. (2018) that the mitotypes (M1, M2 and M3) evolved independently from a common unknown ancestral. 

Scolecophidia mitotypes (M1 and M2) show the lack of the OL region which could be associated with these snakes’ fossorial habits (Miralles et al., 2018). However, this hypothesis should be tested in future studies focusing on the impact of these variations in animal ecology and niche occupancy.

Mitogenome 3 and its derivatives (M3A to M3K) are observed in Alethinophidia, with the M3 as the most frequent and observed in all families, but Tropidophiidae, Viperidae, and Homalopsidae. The prevalence of M3 and the occurrence of mitotypes in specific clades can be hypothesized to be related to diversification rates, snake dispersal during the Paleogene and Neogene (Klein et al., 2021) and later adaptations (e.g. marine lifestyle observed in Hydrophiinae, venomous traits in Viperidae, although these hypotheses are still open to be tested).

The mitogenomic diversity observed herein is associated with rearrangements (duplication and translocation) of non-coding regions or tRNAs, specifically in four clusters: (i) the WAN-Ol-CY; (ii) the CR (I or II) + adjacent tRNA (tRNA-P, tRNA-I, and tRNA-T); (iii) the S2D; and (iv) the IQM. The existence of these duplicates has been proposed for many taxonomic groups and can be partially explained as remnants of ancient rearrangements that vary in arrangements and size, involving overlapping tRNAs (Macey et al., 1997; Pereira, 2000; Bernt et al., 2013b; Xiaokaiti et al., 2022).

Herein, we highlighted that these rearrangements may have led to: (i) the presence of a N-tRNA duplicate (N*) in *Hydrophis cyanocinctus*; (ii) the inversion in the order of the OL region and the N-tRNA in *H. curtus*, *H. melanocephalus*, *H. platurus*, and *Emydocephalus ijimae* as shown by Xiaokaiti et al. (2022); (iii) the presence of an F-tRNA duplicate (F*) and a NC region in *B. insularis*, which was also described for *B. jararaca* by Almeida et al. (2016); (iv) the presence of duplicates of conserved sequence blocks (C-rich, Hairpin II, Core TA, CSB I and CSBIII) on *Bothrops diporus*, *B. pubescens, Daboia ruselli*, *Deinakistrodon acutus*, *Protobothrops himalayanos*, *Pseudoleptodeira latifasciata*, *Typhlops vermiculatus* and *Vipera berus* control regions; (v) the presence of repetitive motifs on *Boa constrictor*, *Emydocephalus ijimae, Hydrophis melanocephalus*, *H. platurus*, *Imantodes cenchoa*, *Leptodeira septentrionalis polysticta* and *Typhlops vermiculatus* control regions; (v) the presence of a Isoleucine-tRNA duplicate vestige (I**) in *Ophiophagus hannah* and the presence of a Isoleucine-tRNA duplicate (I*) in *Hypsiglena jani texana* (New Mexico, USA); (vi) the presence of a Serine 2-tRNA duplicate (S2*) in *H. curtus* and *H. cyanocinctus*; and (vii) the presence of a Arginine-tRNA duplicate (D*) in *Micrurus fulvius*.

Notwithstanding, the original paper that described the mitogenomes of *O. hannah* reported a duplicated tRNA I* sequence (Chen and Lai, 2010), our analysis recovered an additional duplicated vestigial tRNA-I**, that might have appeared before the duplicate tRNA I* and degenerative processes. Furthermore, the description of the *Hypsiglena jani texana* (New Mexico, USA) mitogenome and the revised description of the *Hydrophiinae* mitogenomes were not available when the last snake mitogenome revisions were carried (Qian et al., 2018; Myers and Mulchahy, 2020; Xiaokaiti et al., 2022). Thus, they were also included as reannotated, as a novelty, or cited in this revision.

Several groups of metazoans show variations in the WAN-Ol-CY cluster order (for example, see Mueller and Boore, 2005; Mauro et al., 2006; Fujita et al., 2007; Bernt et al., 2013 a ) and in the CR (I or II) and adjacent tRNA cluster order (for example see Bernt *et al.*, 2013a). In snakes, variations related to these clusters involved the lack of the OL, the translocation of the tRNA-Q, and the presence of duplicates of the tRNA-P (P*), tRNA-I (I*), and tRNA-F(F*) and their translocations (Chen and Zhao, 2009; Almeida et al., 2016; Qian et al., 2018; Myers and Mulchahy, 2020). However, variations in the *S2D* cluster order had not been described in snake mtDNA and are considered as one of our revision novelties.

Although the rearrangements highlighted above occur in different regions of the mitogenomes, their origin may be explained by the errors in the mtDNA replication process, which are associated with the higher mutation rates observed in snake mitogenomes compared to other animal taxa (Kumazawa and Nishida, 1995; Macey et al., 1997; Jiang et al., 2007; Castoe et al., 2009; Bernt et al., 2013 a). These errors may lead to the occurrence of TDRL process followed by the translocation of these duplicates or the original segment, promoting the gene order variations, as observed. This is consistent with previous proposals regarding the duplications of tRNAs and its rearrangements on mitogenomes (Kumazawa *et al.*, 1996; Qian et al., 2018; Xiaokaiti et al., 2022). Furthermore, these tRNAs duplicates can become nonfunctional, due to the accumulation of deleterious mutations over time (Macey *et al.*, 1997). Functional analyses or comparisons of the effectiveness of the acceptor arm may help to understand their evolutionary significance - functional duplicate or a relict segment only - (Mabuchi et al., 2004; Xiaokaiti *et al.*, 2022) and therefore could be a trend topic for future research.

Additionally, control region analysis showed a high conserved pattern among snakes, especially to Caenophidia. The high similarity between the two control regions has been proposed as a result of concerted evolution events (Kumazawa, 2004,) with presence of the conserved sequence blocks and variable usage of each region (Castoe et al., 2009). Duplications of some of these conserved blocks were observed on specific species and might originate from slippage during replication process or TDRL events involving the 5’ initial segments of the control regions, or mitochondria heteroplasmy as proposed by Xiaokaiti et al. (2022). 

Although the original CSB-III sequence location showed divergences, previous annotations of Dipsadidae species describe a 14bp-sequence length similar to CSB-III (Myers and Mulchahy, 2020). After manual curation, we observed that this region was not that similar to CSB-III described earlier, but it is conserved among all snakes analyzed. Then, herein we reported it as the CSB-III-like region (due to revised annotation) and as one of the conserved blocks found in snakes. 

Repetitive sequences were found on initial and terminal parts of control region across different species. Although *Hydrophiinae* repeats were previously described (Xiaokaiti et al., 2022) we highlight that the repeats observed in the control region II of *H. curtus*, and both sequences of *H. melanocephalus* available were composed of the same motif repeated multiple times, suggesting a continuous evolutionary path. Other repeats were previously found in *Boa constrictor* (Kumazawa et al., 1996), *Leptodeira septentrionalis polysticta -* the previous *Leptodeira polysticta* (Myers and Mulchahy, 2020); *Sibon nebulatus* (Myers and Mulchahy, 2020), *Imantodes cenchoa* (Myers and Mulchahy, 2020), *Pseudoleptodeira latifasciata* (Myers and Mulchahy, 2020); though motifs were not characterized until this revision. Notably, the repeats in *Typhlops reticulatus* were never discussed or shown in the literature, representing a finding of this study.

Despite the repetitive nature of the control region had already been established and confirmed by early studies (Kumazawa et al., 1996), the impact of the duplications observed in this study (both conserved blocks and repetitive motifs) remains unknown. One hypothesis would be that duplication could affect the expression of mitogenomic components (Xiaokaiti et al., 2022), however functional studies are necessary to investigate it.

### Implications for conservation

Focusing on the Brazilian species *B. insularis*, the mitogenome structure shows significant differences when compared to other *Bothrops* snakes. Comparison of mitochondrial DNA of *B. insularis* and *B. jararaca* revealed that nucleotide composition differs (98.5% of similarity) - albeit they show similar structure orders - which is similar to the difference between *B. diporus* and *B. pubescens* (98%) mitogenomes. This information associated to morphological, ecological and variation in venom, as well as the absence of gene flow due to the insularization reinforce *B. insularis* as an evolutionary significant unit (ESU) within the Neotropical set of pitvipers, and with biomedical relevance. 

## Conclusion

This study characterized the complete mitogenome of the critically endangered Brazilian snake *Bothrops insularis* (17,523 bp - 39 mitogenomic components). A key diagnostic feature is the duplicate tRNA-Phe and the presence of a non-coding region. Additionally, *Bothrops* species exhibits exclusive mitotypes. Based on this, we hypothesize this genus and others Brazilian species could offer valuable insights into the evolutionary dynamics of snake mitogenomes, and harbor unrecognized mitotypes. We propose new hypothesis regarding snake mitogenome evolution, emphasizing the high mitogenomic diversity in these taxa, especially in Viperidae, Elapidae, and Dipsadidae; and recovered the first ancestral reconstruction state analysis. The remarkable diversity observed can be explained by rearrangements of tRNA clusters and control region segments or by the occurrence of heteroplasmy in the mtDNA of some species. To enhance our comprehension on this phenomenon, we recommend analyzing heteroplasmy by sequencing multiple individuals of the same species to enhance the accuracy of mitogenome analyses and to keep improving the availability and amount of complete mitogenomes. Additionally, we highlight that manual curation with comparisons of previously described sequences and the characterization of this molecule using high-throughput sequencing may help to reduce inaccuracies and improve the comprehension of the repetitive nature of the control region segments. At last, the mitogenome diversity observed raises additional questions to further investigation: (i) Could the organization of mitotypes be related to the ecological habits or habitats of snakes? (ii) How the divergences in control regions affect their usage and expression of mitogenomic contents? These questions are still open to be explored.

## Supplementary Material

The following online material is available for this article:

Table S1 - List of species used in this study.

Table S2 - Mitogenomic components of the *Bothrops insularis* mitogenome.

Figure S1 - Secondary structure predicted for the tRNA sequences of the *Bothrops insularis* mitogenome.

## Data Availability

*Bothrops insularis* mitogenome described and analyzed in this study is available at the NCBI under accession number PX647012.
