# Supplementary material for: Structural evolution of the whole mitochondrial genome and phylogenetic inference in snakes (Squamata: Serpentes), including the undescribed mitogenome of the Brazilian endemic and critically endangered pitviper Bothrops insularis
Source: Genet Mol Biol. 2026 Jan 30;48(4):e20240196. doi: 10.1590/1678-4685-GMB-2024-0196 (PMC12893195; doi:10.1590/1678-4685-GMB-2024-0196)
Supplement: Table S1 - [file 1415-4757-GMB-48-04-e20240196-s1.pdf]

Genetics and Molecular Biology
https://doi.org/10.1590/1678-4685-GMB-2024-0196

Supplementary Material to "Structural evolution of the whole mitochondrial genome and phylogenetic inference in snakes (Squamata: Serpentes), including the undescribed mitogenome of the Brazilian endemic and critically endangered pitviper *Bothrops insularis*"

Table S1 – List of species used in this study.

Infraorder	Hyperfamily	Family	Genus	Species	GenBank Access number	Length (bp)	Reference
Alethinophidiai	Caenophidia	Acrochordidae	*Achochordus*	*granulatus*	NC007400	17604	Dong & Kumazawa, 2005.
		Colubridae	*Elaphe*	*anomala*	NC027001	17164	Liu & Zhao, 2016.
				bimaculata	NC024743	17183	Yan *et al*., 2016.
				carinata	KU180459	17154	Ding *et al*., 2016.
				davidi	NC025643	17117	Xu *et al*., 2016.
				dione	NC041068	17172	Simonov *et al*., 2018.
				quatuorlineata	MK334307	17183	Jablonski *et al*., 2019.
				sauromates	MK070315	17187	Jablonski*et al*., 2019.
				schrenckii	NC027605	17165	Liu & Zhao, 2016.
				taeniura	NC025275	17183	Li *et al*., 2016.
			Euprepiophis	*perlacea*	NC024546	17160	Wan *et al*., Unpublished.
			Lycodon	*flavozonatus*	NC028730	17172	Ding *et al*., 2016.
				rufozonatus	NC024559	17188	Qian *et al*., 2018.
				ruhstrati	NC046046	17168	Gong *et al*., 2019.
				semicarinatus	NC001945	17191	Kumazawa *et al*., 1998.
			Oocatochus	*rufodorsatus*	NC022146	17159	Li *et al*., 2014.
			Oreocryptophis	*porphyraceus*	NC012770	17167	Lin *et al*., Unpublished.
			Orientocoluber	*spinalis*	NC049067	17196	Park *et al*., Unpublished.
			Pantherophis	*guttatus*	AM236349	16445	Douglas *et al*., 2006.
				slowinskii	NC009769	17189	Jiang *et al*., 2007.
			Pituophis	*catenifer sayi*	KU833245	17193	Lele *et al*., 2016.
			Ptyas	*dhumnades*	NC028049	17164	Li *et al*., Unpublished
				major	NC028048	17217	Sun *et al*., 2017.
				mucosa	NC030041	17151	Zhou *et al*., 2016.
			Stichophanes	*ningshaanensis*	NC026083	17292	Wang & Zhu, Unpublished.

Infraorder	Hyperfamily	Family	Genus	Species	GenBank Access number	Length (bp)	Reference
		Natricidae	*Hebius*	*optatum*	MN427890	17259	Zong *et al*., 2020.
				vibakari ruthveni	KP684155	17259	Xu *et al*., 2016.
			Nerodia	*sipedon*	NC015793	17706	Huff *et al*., Unpublished.
			Opisthotropis	*latouchii*	NC046823	17051	Wang *et al*., 2019.
			Rhabdophis	*tigrinus*	NC030210	17415	Zhao *et al*., 2016.
		Sibynophiidae	*Sibynophis*	*chinensis*	NC022430	17163	Oh *et al*., 2015.
				collaris	NC016424	17163	Jang & Hwang, 2011.
		Dipsadidae	*Hypsiglena*	*affinis*	MT561499	17190	Myers & Mulchahy, 2020.
				catalinae	KJ486459	17208	Mulcahy *et al*., 2014.
				chlorophaea chlorophaea	NC013977	17209	Mulcahy & Macey, 2009.
				chlorophaea deserticola	NC013989	17209	Mulcahy & Macey, 2009.
				jani jani	MT561500	17205	Myers & Mulchahy, 2020.
				jani texana (Texas, USA)	NC013975	17203	Mulcahy & Macey, 2009.
				jani texana (New Mexico, USA)	MT561497	17235	Myers & Mulchahy, 2020.
				ochrorhyncha klauberi	NC013984	17205	Mulcahy & Macey, 2009.
				ochrorhycha nuchalata	NC013983	17207	Mulcahy & Macey, 2009.
				ochrorhyncha ochrorhyncha	NC013980	17205	Mulcahy & Macey, 2009.
				slevini	NC013987	17197	Mulcahy & Macey, 2009.
				sp. *(Arizona, USA)*	MT561495	17200	Myers & Mulchahy, 2020.
				sp. *(Arizona, USA)*	NC013982	17205	Mulcahy & Macey, 2009.
				torquata	NC013992	17203	Mulcahy & Macey, 2009.
				unaocularus	NC024164	17211	Mulcahy *et al*., 2014.
			Imantodes	*cenchoa*	NC013988	22144	Mulcahy & Macey, 2009.
			Leptodeira	*septentrionalis polysticta*	NC013990	17506	Mulcahy & Macey, 2009.
			Pseudoleptodeira	*latifasciata*	NC013981	18329	Mulcahy & Macey, 2009.
			Sibon	*nebulatus*	NC013985	22887	Mulcahy & Macey, 2009.
			Thermophis	*baileyi*	NC035713	17355	Sun, 2017.
				shangrila	NC035058	17407	Wu *et al*., 2017.
				zhaoermii	NC012816	17322	He *et al*., 2010.
		Elapidae	*Bungarus*	*fasciatus*	NC011393	17234	Chen & Zhao, 2009.
				multicinctus	NC011392	17144	Chen & Zhao, 2009.
			Micrurus	*fulvius*	NC013481	17506	Castoe *et al*., 2009.
			Naja	*atra*	NC011389	17216	Chen & Zhao, 2009.
				kaouthia	LC431744	17203	Singchat *et al*., 2019.
				naja	NC010225	17213	Yan *et al*., 2008.
			Ophiophagus	*hannah*	NC011394	17267	Chen & Lai, 2010.
			Laticauda	*colubrina*	NC036054	17450	Kim *et al*., 2018.

Infraorder	Hyperfamily	Family	Genus	Species	GenBank Access number	Length (bp)	Reference
				laticaudata	NC036053	17209	Kim *et al*., 2018.
				semifasciata	NC036055	17170	Kim *et al*., 2018.
			Sinomicrurus	*macclellandi*	MT547176	17120	Yao *et al*., 2020.
		Homalopsidae	*Hypsiscopus*	*plumbea*	NC010200	17397	Yan *et al*., 2008.
		Hydrophiidae	*Emydocephalus*	*ijimae*	MT547176	18259	Yi *et al*., 2019.
			Hydrophis	*curtus*	NC046794	17702	Qiu *et al*, 2019.
				cyanocinctus	NC046795	17750	Qiu *et al*., 2019.
				melanocephalus	MK775532	17182	Yi *et al*., 2019.
				platurus	MK775530	18101	Kim *et al*., 2020.
		Viperidae	*Azemiops*	*feae*	NC030781	17383	Geng & Yan, Unpublished.
			Agkistrodon	*contortrix*	NC035638	16269	Xu & Yu, 2017.
				piscivorus	NC009768	17213	Jiang *et al*., 2007.
			Bothrops	*diporus*	NC039649	17642	Kleiz *et al*., Unpublished.
				insularis	PX647012	17523	This study
				pubescens	NC039648	17694	Kleiz *et al*., Unpublished.
				jararaca	NC030760	17526	Almeida *et al*., 2016.
			Crotalus	*adamanteus*	NC041524	17242	Wu, 2019.
				horridus	NC014400	17260	Hall *et al*., 2013.
			Deinagkistrodon	*acutus*	NC010223	17548	Yan *et al*., 2008.
			Gloydius	*brevicauda*	NC011390	17227	Chen & Zhao, 2009.
				intermedius	NC025560	17226	Xu *et al*., 2016.
				saxatilis	NC025666	17218	Xu *et al*., 2016.
				shedaeoensis	NC029424	17222	Liu *et al*., 2016.
				strauchii	NC036234	17224	Yang *et al*., Unpublished.
				ussuriensis	NC026553	17208	Han *et al*., 2016.
			Ovophis	*okinavensis*	NC007397	17388	Dong & Kumazawa, 2005.
			Protobothrops	*cornutus*	NC022695	17219	Zhang *et al.,* 2015.
				dabieshanensis	NC022473	17193	Huang *et al*., 2014.
				flavoviridis	NC030181	17232	Shibata *et al*., 2016.
				himalayanus	NC029165	17389	Huang & Zang, Unpublished.
				jerdonii	NC021402	17239	Huang *et al*., 2013.
				kaulbacki	NC029166	17237	Huang & Zang, Unpublished.
				mangshanensis	NC026052	17230	Huang *et al*., 2014.
				maolanensis	NC026051	17228	Huang *et al*., 2014.
				mucrosquamatus	NC021412	17234	Zhang *et al*., 2013.
				mucrosquamatus	KT447436	17223	Chen *et al*., Unpublished.
				tokarensis	NC030182	17233	Shibata *et al*., 2016.

Infraorder	Hyperfamily	Family	Genus	Species	GenBank Access number	Length (bp)	Reference
				xiangchengsis	KF460436	17240	Zhang *et al*., 2015.
			Trimeresurus	*albolabris*	NC022820	17220	Song *et al*., 2015.
				erythrurus	NC045531	17225	Vaishnavi *et al*., Unpublished.
				sichuanensis	NC029494	17225	Zhu *et al*., 2016.
				stejnegeri stejnegeri	NC012146	17239	Lin *et al*., Unpublished.
			Causus	*defilippii*	NC013479	17342	Castoe *et al*., 2009.
			Daboia	*russellii*	NC011391	17246	Chen & Zhao, 2009.
			Macrovipera	*schweizeri*	NC044966	17152	Thanou & Kornilios, 2019.
			Vipera	*berus*	NC036956	16370	Gao *et al*., 2018.
		Xenodermidae	*Achalinus*	*meiguensis*	NC011576	17239	Wang *et al*., 2009.
				rufescens	NC032085	17339	Zhang *et al*., 2017.
				spinalis	NC032084	17165	Peng *et al*., 2017.
	Henophidia	Boidae	*Boa*	*constrictor*	NC007398	18905	Dong & Kumazawa, 2005.
				imperator	AM236348	16607	Douglas *et al*., 2006.
			Eunectes	*notaeus*	AM236347	17970	Douglas *et al*., 2006.
			Eryx	*tataricus*	MN046174	17537	Cai *et al*., 2020.
		Pythonidae	*Malayopython*	*reticulatus*	NC042397	17641	He, Unpublished.
			Python	*bivittatus*	NC021479	17617	Liu *et al*., Unpublished.
			Python	*molurus molurus*	NC015812	17258	Dubey *et al*., 2012.
			Python	*regius*	NC007399	17245	Dong & Kumazawa, 2005.
		Xenopeltidae	*Xenopeltis*	*unicolor*	NC007402	18872	Dong & Kumazawa, 2005.
		Cylindrophiidae	*Cylindrophis*	*ruffus*	NC007401	17499	Dong & Kumazawa, 2005.
		Aniliidae	*Anilius*	*scytale*	NC014343	17713	Castoe *et al*., 2009.
		Tropidophiidae	*Tropidophis*	*haetianus*	NC012573	17138	Castoe *et al*., 2009.
Scolecophidia	Scolecophidia	Gerrhopilidae	*Gerrhopilus*	*mirus*	AM236345	14662	Douglas *et al*., 2006.
		Leptotyphlopidae	*Rena*	*humilis*	NC005961	16218	Kumazawa, 2004.
		Typhlopidae	*Typhlops*	*reticulatus*	NC010971	16681	Castoe *et al*., 2008.
			Anilios	*australis*	AM236346	14356	Douglas *et al*., 2006.
			Indotyphlops	*braminus*	NC010196	16397	Yan *et al*., 2008.
			Xerotyphlops	*vermicularis*	NC044967	16568	Kornilios *et al*., 2019.
