# Supplementary material for: Structural evolution of the whole mitochondrial genome and phylogenetic inference in snakes (Squamata: Serpentes), including the undescribed mitogenome of the Brazilian endemic and critically endangered pitviper Bothrops insularis
Source: Genet Mol Biol. 2026 Jan 30;48(4):e20240196. doi: 10.1590/1678-4685-GMB-2024-0196 (PMC12893195; doi:10.1590/1678-4685-GMB-2024-0196)
Supplement: Table S2 - [file 1415-4757-GMB-48-04-e20240196-s2.pdf]

Genetics and Molecular Biology
https://doi.org/10.1590/1678-4685-GMB-2024-0196

Supplementary Material to "Structural evolution of the whole mitochondrial genome and phylogenetic inference in snakes (Squamata: Serpentes), including the undescribed mitogenome of the Brazilian endemic and critically endangered pitviper *Bothrops insularis*"

Table S2 – Mitogenomic components of the *Bothrops insularis* mitogenome.

Feature	Position	Length (bp)	Codon		tRNA	Strand
			Start	*Stop*	*Anticodon*	
tRNA-Phe (F)	1 – 65	65			GAA	-
tRNA-Phe (F*)	299 – 362	64			GAA	-
12S	363 – 1279	917				-
tRNA-Val (V)	1280 – 1342	63			UAC	-
16S	1343 – 2822	1480				-
ND1	2823 – 3783	961	ATA	T		-
tRNA-Ile (I)	3784 – 3852	69			GAU	-
tRNA-Pro (P)	3853 – 3920	65			UGG	+
Control Region II (CRII)	3906 – 4938	1033				
tRNA-Leu^{UAA} (L1)	4946 – 5018	73			UAA	-
tRNA-Gln (Q)	5019 – 5088	70			UUG	+
tRNA-Met (M)	5089 – 5151	63			CAU	-
ND2	5152 – 6181	1030	ATT	T		-
tRNA-Trp (W)	6182 – 6249	68			UCA	-
tRNA-Ala (A)	6250 – 6313	64			UGC	+
tRNA-Asn (N)	6314 – 6388	75			GUU	+
Light strand replication origin (O_L)	6391 – 6425	35				
tRNA-Cys (C)	6424 – 6483	60			GCA	+
tRNA-Tyr (Y)	6484 – 6544	61			GUA	+
COX1	6546 – 8147	1602	GTG	AGA		-
tRNA-Ser^{UGA} (S2)	8138 – 8205	68			UGA	+
tRNA-Asp (D)	8206 – 8268	63			GUC	-
COX2	8269 – 8953	685	ATG	T		-
tRNA-Lys (K)	8954 – 9016	63			UUU	-
ATP8	9017 – 9181	165	ATG	TAA		-
ATP6	9172 – 9852	681	ATG	TAA		-
COX3	9852 – 10635	784	ATG	T		-
tRNA-Gly (G)	10636 – 10696	61			UCC	-
ND3	10697 – 11039	343	ATC	T		-
tRNA-Arg (R)	11040 – 11104	65			UCG	-
ND4-L	11105 – 11395	291	ATG	TAA		-
ND4	11395 – 12732	1338	ATG	AGA		-
tRNA-His (H)	12734 – 12795	62			GUG	-
tRNA-Ser^{GCU} (S1)	12796 – 12851	56			GCU	-
tRNA-Leu^{UAG} (L2)	12852 – 12924	73			UAG	-
ND5	12925 – 14712	1788	ATG	TAA		-
ND6	14708 – 15229	522	ATG	AGA		+
tRNA-Glu (E)	15231 – 15293	63			UUC	+
CYTB	15293 – 16406	1114	ATG	T		-
tRNA-Thr (T)	16407 – 16470	64			UGU	-
Control region I (CRI)	16471 – 17523	1030				

(+) = Heavy strand. (-) = Light strand.
