# Supplementary material for: Structural evolution of the whole mitochondrial genome and phylogenetic inference in snakes (Squamata: Serpentes), including the undescribed mitogenome of the Brazilian endemic and critically endangered pitviper Bothrops insularis
Source: Genet Mol Biol. 2026 Jan 30;48(4):e20240196. doi: 10.1590/1678-4685-GMB-2024-0196 (PMC12893195; doi:10.1590/1678-4685-GMB-2024-0196)
Supplement: Figure S1 - [file 1415-4757-GMB-48-04-e20240196-s3.pdf]

Genetics and Molecular Biology
https://doi.org/10.1590/1678-4685-GMB-2024-0196

Supplementary Material to "Structural evolution of the whole mitochondrial genome and phylogenetic inference in snakes (Squamata: Serpentes), including the undescribed mitogenome of the Brazilian endemic and critically endangered pitviper *Bothrops insularis*"

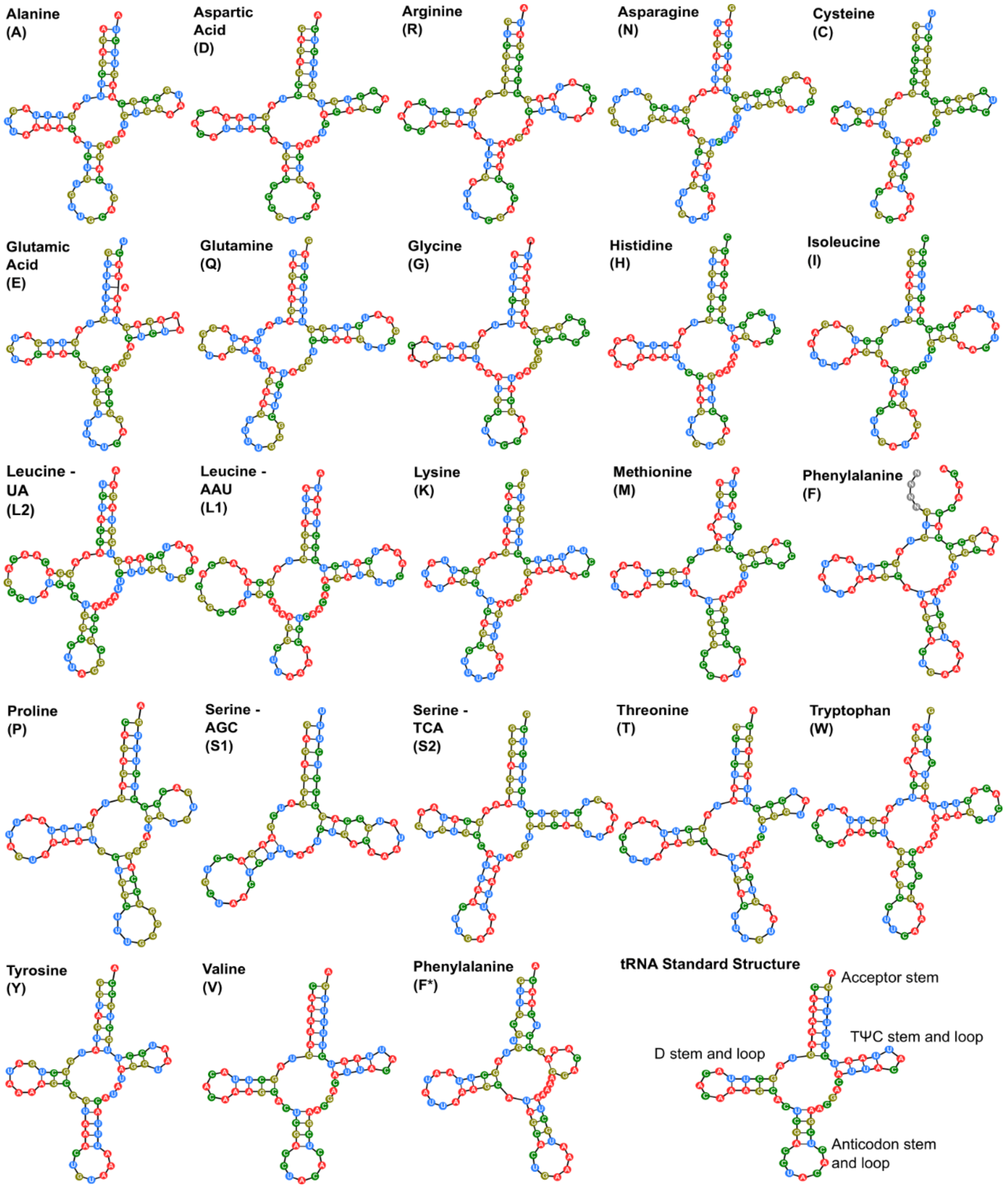

Figure S1 – Secondary structures predicted for tRNA sequences of the *Bothrops insularis* mitogenome.

Red circles = Adenine (A). Green = Cytosine (C). Brown = Guanine (G). Blue = Uracil (U).
